# Supplementary material for: ThermoSlope: A Software for Determining Thermodynamic Parameters from Single Steady-State Experiments
Source: Molecules. 2021 Nov 26;26(23):7155. doi: 10.3390/molecules26237155 (PMC8658824; doi:10.3390/molecules26237155)
Supplement: Supplementary file 1 [file molecules-26-07155-s001.zip › molecules-1462376-supplementary.pdf]

ThermoSlope: a software for determining thermodynamic parameters from single steady-state experiments

Bjarte Aarmo Lund [1,*] and Bjørn Olav Brandsdal [1]

[1] Hylleraas Centre for Quantum Molecular Sciences, Department of Chemistry, Faculty of Science and Technology, UiT The Arctic University of Norway, N9037 Tromsø, Norway
* Correspondence: Bjarte.lund@uit.no; Tel.: +4777646477

Supplementary information

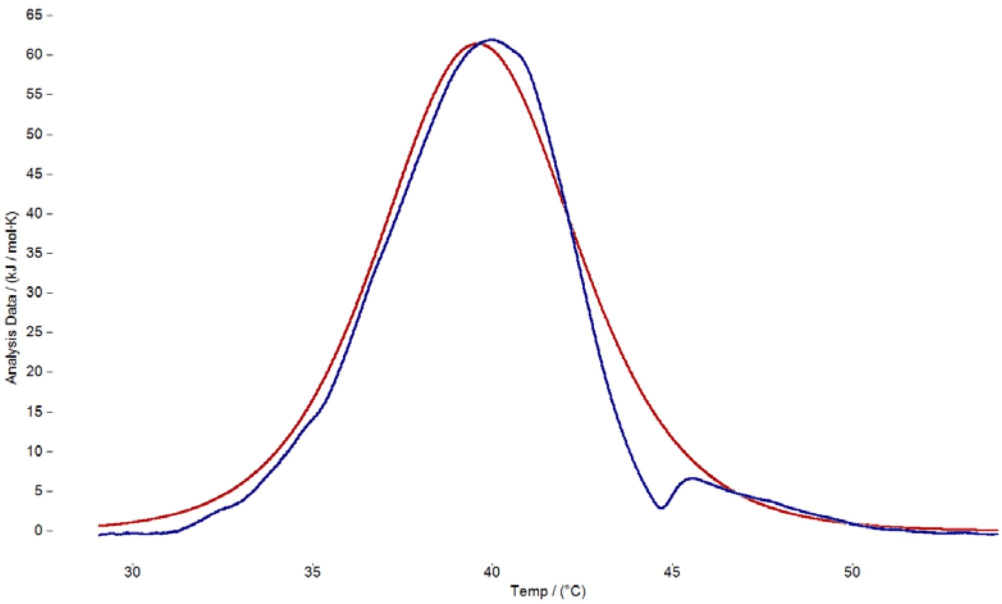

Figure S1: The differential scanning calorimetry curves (blue) for the thermal unfolding of pLipA reveals a single event that fits well to a two-state model (red). The fitted Tm is 39.6°C and the area under the curve corresponds to a ΔH of 447 kcal/mol.
